# Genetic Alterations in Benign Breast Biopsies of Subsequent Breast Cancer Patients

**DOI:** 10.3389/fmed.2019.00166

**Published:** 2019-07-24

**Authors:** Savas D. Soysal, Charlotte K. Y. Ng, Luigi Costa, Walter P. Weber, Viola Paradiso, Salvatore Piscuoglio, Simone Muenst

**Affiliations:** ^1^Clarunis, University Hospital Basel, Basel, Switzerland; ^2^Visceral Surgery Research Laboratory, Department of Biomedicine, Clarunis, Basel, Switzerland; ^3^Institute of Pathology, University Hospital Basel, Basel, Switzerland; ^4^Department for BioMedical Research, University of Bern, Bern, Switzerland; ^5^Breast Center, University Hospital Basel, Basel, Switzerland

**Keywords:** breast cancer, genetic alteration, fibrocystic mastopathy, carcinogenesis, somatic mutation

## Abstract

**Background:** Fibrocystic changes are associated with an increased risk of breast cancer. Genetic alterations have been found in fibrocystic changes with or without epithelial changes, suggesting that critical oncogenic events are occurring at an early stage.

**Methods:** We investigated a unique collective of 17 breast cancer patients who, prior to the diagnosis of invasive breast cancer, underwent open surgical biopsy showing fibrocystic changes of the breast. The time span between biopsy for fibrocystic changes and invasive carcinoma ranged from 1 to 11 years (average 5.3 years). Ten (58.8%) of the patients had an ipsilateral invasive carcinoma, and 7 (41.2%) of the patients developed an invasive carcinoma of the contralateral breast. Massive parallel sequencing targeting genes frequently mutated in breast cancer was performed on the fibrocystic breast tissue as well as the ensuing cancer tissue.

**Results:** In 9 cases, somatic mutations were found in the tumor tissue, the most prevalent being *PIK3CA* mutations (*n* = 4), followed by *TP53* mutations (*n* = 2). None of these mutations were present in the previously removed mastopathy tissue. In one of the cases, an *ERBB3* E928G mutation was present in the mastopathy as well as in the tumor tissue, with the variant allele frequency in the mastopathy being <0.1%. In two patients, we found two mutations (*MAP3K1* L380fs and *PIK3CA* I391M, respectively) present in the mastopathy as well as in the subsequent breast cancer. These two mutations, however, could also be due to fixation artifacts.

**Conclusion:** Since no significant somatic mutations in the fibrocystic breast tissue, and only doubtful shared mutations between benign and associated cancer tissue were detected, it remains unclear why women with fibrocystic breast disease have a statistically significant increased risk of breast cancer. Further analyses, maybe on the level of gene expression, could help to clarify the role of these benign alterations in the development of breast cancer and help to identify women at greater risk of developing subsequent invasive cancer.

## Introduction

Breast cancer continues to be the most common cancer in women and represents a major public health issue with 1.38 million new cases and almost half a million deaths yearly worldwide (1). Since primary prevention of breast cancer is still not available, efforts to promote early detection continue to be the major focus in fighting this type of tumor, given that early detection is associated with decreased mortality (2).

Fibrocystic changes of the breast, also called fibrocystic mastopathy, is a very common benign disease, by which up to 50% of premenopausal women are affected (3). Fibrocystic mastopathy encompasses several histopathologic changes such as cyst formation, apocrine metaplasia, papillomatosis, duct ectasia, sclerosing adenosis, and stromal fibrosis (4). These changes are often accompanied by epithelial changes such as usual ductal hyperplasia (UDH), flat epithelial atypia (FEA), and benign columnar cell lesions (CCL) (5). Classic fibrocystic changes with or without epithelial changes are not regarded as a precancerous lesion, since only a small minority of these women develop invasive carcinoma. However, women with fibrocystic breast disease have a significantly increased risk of breast cancer (~1.5–2 times that of the general population), which is especially evident in women 50 years of age and older, and is independent of other key epidemiologic breast cancer risk (6–8). Although the relative risk seems low, because of the high frequency of such changes in the general population, the absolute risk is significant. However, predicting which patients will progress to invasive cancer remains difficult, a fact that motivates research aimed at uncovering the molecular mechanisms at play in these earliest stages of cancer development (5).

Numerous studies have found genetic aberrations in histologically normal breast tissue of patients with breast cancer, indicating a mammary field cancerization (9–11), and the widely accepted model of breast cancer oncogenesis is that it develops from benign breast tissue through a succession of genetic alterations, leading first to *in-situ* and subsequently to invasive lesions (12). A recent study using whole-genome sequencing showed that epithelial changes such as UDH, FEA, and CCL have already acquired a significant number of genomic alterations, such as point mutations and chromosome aneuploidies, suggesting that critical oncogenic events are occurring at this early stage (13). Many of these alterations are observed in both the patient's benign lesions and associated invasive cancer, thus definitively establishing a genetic relationship. These molecular alterations are one of the earliest events that affect a large number of genes and may provide the initial oncogenic potential and help trigger clonal expansion of imminent breast cancer cells (13).

In a previous study by our group, FISH analysis revealed that the estrogen receptor 1- gene (*ESR1)* was amplified in 15.5% of breast cancers. Interestingly, women with *ESR1* amplification in breast cancer displayed this amplification even in the benign fibrocystic breast tissue prior to the first diagnosis of cancer, and this amplification was absent in fibrocystic tissues from women who did not develop breast cancer (14). This suggests that molecular alterations such as *ESR1* gene amplifications are an early event in breast carcinogenesis and are at least in part already present in fibrocystic breast disease.

The aim of our study was to investigate if patients that subsequently develop invasive carcinoma of the breast already exhibit molecular alterations in fibrocystic breast tissue removed years earlier. For this, we performed targeted massive parallel sequencing on fibrocystic breast tissue of women with subsequent invasive breast cancer.

## Materials and Methods

### Identification of Patients

We performed an in-depth search of the archive of the Institute for Pathology for matched specimens from breast cancer patients. We identified 53 patients with surgically removed invasive breast cancer. Prior to the breast cancer diagnosis, all patients had undergone open surgical biopsy where fibrocystic changes of the breast were diagnosed. None of the patients had an invasive carcinoma, atypical ductal hyperplasia (ADH) or ductal or lobular carcinoma *in situ* (DCIS and LCIS) in these prior biopsies. For all patients, a normal control sample (lymph node, skin, or gastric biopsy) was also identified.

The study was conducted in concordance with institutional patient safety laws and has been approved by the Ethikkommission Nordwest- und Zentralschweiz (EKNZ, proposal number 2014-397).

### Identification of Appropriate Tissue

H&E slides and formalin-fixed, paraffine-embedded (FFPE) tissue blocks were retrieved from the archives of the Institute of Pathology. Where needed, fresh H&E slides were obtained. All biopsies underwent reevaluation by a certified breast pathologist (SM) in order to confirm the diagnosis of fibrocystic breast disease with or without UDH, FEA, or CCL. Cases with atypical ductal hyperplasia (ADH) or ductal or lobular carcinoma *in situ* (DCIS and LCIS) were excluded. Concordantly, all of the subsequent carcinomas were reevaluated and confirmed. Subsequently, suitable regions for genomic analyses were identified on the H&E slides. Since fibrocystic breast disease consists of glandular structures and cysts as well as a considerable amount of fatty and/or collagenous tissue, dilution of the epithelial DNA of interest was minimal. For the invasive breast cancer, a tumor cell content of >70% was considered sufficient for DNA analysis. For each patient, normal tissue such as lymph nodes or gastric biopsies were also sequenced as germline control. Fresh uncovered H&E slides were cut from the formalin-fixed and paraffin-embedded tissue. The suitable tissue was mechanically scraped off, or, in case of few epithelial cells, removed by laser microdissection to ensure a high purity of the analyzed cells.

### Design of Breast Cancer Gene Panel

A custom targeted sequencing panel focusing on the most frequently altered genes in breast cancer was designed using Ion Ampliseq Designer (https://www.ampliseq.com/browse.action; Thermo Fisher Scientific, MA, USA). The panel covers all exons of 27 protein-coding genes as well as mutation hotspots in three cancer genes are also covered, and the recurrently mutated lncRNA genes *MALAT1* and *NEAT1* (Supplementary Table 1) (15). The panel was designed using the FFPE option for smaller amplicon size. Notably, our primary interest was not the screening for novel mutations; instead focused on a specific panel of already described breast cancer-relevant mutations and designed a mutation panel encompassing the most relevant genes.

### DNA Extraction and Library Preparation

For each of the patients enrolled in this study, genomic DNA was extracted from the benign fibrocystic breast tissue and from the tumor biopsies. DNA extracted from a biopsy of normal tissue, such as lymph nodes or gastric mucosa, served as germline control. Briefly, representative formalin-fixed paraffin-embedded histologic blocks were stained with Nuclear Fast Red in RNase-free condition and subjected to microdissection with a sterile needle under a stereomicroscope (Olympus) to ensure a percentage of tumor cells >70%, as described previously (16). DNA was extracted with the DNeasy Blood and Tissue Kit (Qiagen, Hilden, Germany) according to manufacturer's instructions as previously described (17, 18). DNA was quantified using the Qubit Fluorometer (Thermo Fisher Scientific).

Library preparation for the breast panel was performed using the Ion AmpliSeq library kit 2.0 (Thermo Fisher Scientific) according to the manufacturer's guidelines. The panel consists of two pools of amplification primers. Ten nanogram of DNA per sample was used for library preparation for each pool. Amplification was performed according to the manufacturer's guidelines. The amplicons from the two pools were combined and treated to digest the primers and to phosphorylate the amplicons. The amplicons were then ligated to Ion Adapters (Thermo Fisher Scientific) using DNA ligase. Finally, cleaning and purification of the generated libraries were performed with Agencourt AMPure XP (Beckman Coulter, CA, USA) according to the manufacturer's guidelines. Quantification and quality control were performed with Ion Library TaqMan Quantitation Kit (Thermo Fisher Scientific). Samples were diluted to reach the concentration of 40 pmol and then were pooled for sequencing. Twenty five microliter of the pooled libraries was loaded on Ion 530 Chip (Thermo Fisher Scientific) and processed in Ion Chef Instrument (Thermo Fisher Scientific). Sequencing was performed on Ion S5 XL system (Thermo Fisher Scientific) (18).

### Sequence Data Analysis

Sequence reads were aligned to the human reference genome hg19 using TMAP within the Torrent Suite Software 5.4 for the Ion S5XL system (17, 18). Somatic mutations were identified using Torrent Variant Caller v5.0.3 (Thermo Fisher Scientific, MA, USA). Mutations at hotspot residues were white-listed (19, 20). We filtered out mutations with quality score <60, and/or supported by <8 reads, and/or those covered by <10 reads in the tumor or <10 reads in the matched non-tumoral counterpart (17, 18). Only those for which the tumor variant allele fraction (VAF) was >10 times that of the matched non-tumoral VAF were retained to ensure the somatic nature of the variants (17, 18). To further ensure that the variant calls were not fixation artifacts, we further classified the variants into confidence tiers based on (1) whether the mutation affected hotspot residues, (2) VAF and read depth in both the tumor/mastopathy and germline samples, (3) whether the variant resulted in C>T/G>A substitution that is typical of fixation artifacts, (4) whether the variant was a homopolymeric insertion/deletion, and (5) whether the variant was supported by reads from >1 amplicon. Tier 1 mutations were considered likely genuine mutations, while tier 2 mutations showed features suggestive of fixation artifact. To account for somatic mutations that may be present at low VAF in either the tumor biopsy or the mastopathy but not both, all somatic mutations identified in one of the two samples were interrogated for their presence in the matched sample by supplying TVC with their positions as the “hotspot list” (17). All reported mutations were manually inspected using the Integrative Genomics Viewer.

## Results

Due to fixation artifacts and the age of the tissue samples, we were only able to perform matched sequencing of mastopathy, control and cancer tissues on 17 of the 53 identified patients (32%). In the other cases, the mastopathy or the control tissue was qualitatively not good enough to be sequenced. For these 17 sequenced patients, the time span between biopsy for fibrocystic changes and invasive carcinoma ranged from 1 to 11 years (average 5.3 years, Table 1). Ten (58.8%) of the patients had an ipsilateral invasive carcinoma, and 7 (41.2%) of the patients developed an invasive carcinoma of the contralateral breast. Twelve of the breast cancers (70.5%) were of Luminal A subtype, two (12%) had a Luminal B subtype, and one of the breast cancers was of Luminal B, Her2 positive subtype. For two patients, the intrinsic subtypes were not available. Regarding the histologic subtypes, the majority (14 of 17 patients, 82%) of the tumors were invasive ductal carcinomas, with two invasive lobular and one tubule-lobular subtype, respectively. Twelve patients (70.5%) presented with stage pT1, and 5 patients (29.5%) presented with stage pT2. Six of the patients (35%) had lymph node metastases at the time of surgery. Clinicopathological information can be found in Table 1.

**Table 1:** Clinicopathological data of the sequenced breast cancer patients.

**Patient**	**Age at cancer diagnosis (years)**	**Localization of cancer and mastopathy**	**Time between mastopathy biopsy and cancer diagnosis (years)**	**Histological subtype**	**TNM classification**	**Intrinsic subtype**
1	67	Contralateral	5	Invasive ductal	pT1c, pN0, G2	Luminal B
2	67	Contralateral	3	Invasive ductal	pT1c, pN0; G2	Luminal B
3	39	Ipsilateral	6	Invasive ductal	pT2, pN1a, G2	NA
4	46	Contralateral	6	Invasive ductal	pT2, pN0, G1	Luminal A
5	49	Contralateral	6	Invasive ductal	pT1b, pN0, G3	Luminal A
6	65	Ipsilateral	11	Invasive lobular	pT1b, pN1a, G2	Luminal A
7	50	Ipsilateral	5	Invasive ductal	pT1c, pN0, G2	Luminal A
8	57	Ipsilateral	7	Invasive ductal	pT1c, pN1mi, G2	Luminal A
9	48	Ipsilateral	5	Invasive ductal	pT1c, pN0, G3	NA
10	48	Ipsilateral	1	Invasive lobular	pT2, pN2, G2	Luminal A
11	61	Ipsilateral	7	Tubulo-lobular	pT1c, pN0, G2	Luminal A
12	58	Ipsilateral	3	Invasive ductal	pT2, pN2a	Luminal A
13	60	Contralateral	5	Invasive ductal	pT1a, pN0, G2	Luminal A
14	59	Contralateral	3	Invasive ductal	pT2, pN3a, G2	Luminal B, Her2 positive
15	58	Ipsilateral	6	Invasive ductal	pT1c, pN0, G2	Luminal A
16	61	Contralateral	7	Invasive ductal	pT1b, pN0, G1	Luminal A
17	76	Ipsilateral	4	Invasive ductal	pT1, pN0, G1	Luminal A

In 9 cases, somatic mutations were found in the tumor tissue, including hotspot mutations in *PIK3CA* (*n* = 4) and *TP53* (*n* = 2, Table 1). None of these hotspot mutations were present in the previously removed mastopathy tissue, despite achieving an average sequencing depth of 2516x (range 814x−5654x). We further identified an additional 15 mutations in *PIK3CA, MAP3K1, GATA3, ERBB3, ARID1A, PIK3R1*, and *KMT2C*, most of which were not detected in the corresponding mastopathies. In one of the cases, an *ERBB3* E928G mutation was present in the mastopathy as well as in the tumor tissue, although the variant allele frequency in the mastopathy was <0.1%. In one case, a *MAP3K1* L380fs mutation was present in the mastopathy as well as in the tumor tissue, and in another case, a *PIK3CA* I391M mutation was present in the mastopathy as well as in the subsequent breast cancer. These last two mutations, however, could also be due to fixation artifacts. The detected mutations for each patient can be found in Table 2. Sequencing data are available at the European Genome-phenome Archive under the accession EGAS00001003563.

**Table 2:** Somatic mutations detected in mastopathy and subsequent breast cancer.

**Patient**	**Mutations tier 1 confidence**	**Mutations tier 2 confidence**
1		Tumor: MAP3K1 L380 PRESENT, MAP3K1 P1474fs PRESENT, PIK3CA stop lost PRESENT Mastopathy: MAP3K1 L380 PRESENT, MAP3K1 P1474fs ABSENT, PIK3CA stop lost ABSENT
2	Tumor: GATA3 A333fs PRESENT Mastopathy: GATA3 A333fs ABSENT	Tumor: FBXW7 c.1122+1G>A PRESENT Mastopathy: FBXW7 c.1122+1G>A ABSENT
3		
4		
5		
6	Tumor: ERBB3 E928G PRESENT, PIK3CA stop lost PRESENT Mastopathy: ERBB3 E928G PRESENT (<0.1% VAF), PIK3CA stop lost ABSENT	
7	Tumor: TP53 I195T PRESENT Mastopathy: TP53 I195T ABSENT	
8	Tumor: KMT2C (MLL3) c.590+1G>C PRESENT Mastopathy: KMT2C (MLL3) c.590+1G>C ABSENT	
9		Tumor: GATA3 S428fs PRESENT Mastopathy: GATA3 S428fs ABSENT
10	Tumor: PIK3CA C378Y PRESENT Mastopathy: PIK3CA C378Y ABSENT	
11		Tumor: ARID1A G444S PRESENT Mastopathy: ARID1A G444S ABSENT
12	Tumor: PIK3CA H1047R PRESENT Mastopathy: PIK3CA H1047R ABSENT	Tumor: PIK3CA I391M present Mastopathy: PIK3CA I391M present
13		
14	Tumor: PIK3CA E545G, TP53 R248W, PIK3C1 K567_L570del PRESENT Mastopathy: PIK3CA E545G, TP53 R248W, PIK3C1 K567_L570del ABSENT	
15	Tumor: KMT2C (MLL3) C1004F PRESENT Mastopathy: KMT2C (MLL3) C1004F ABSENT	
16	Tumor: PIK3CA H1047R PRESENT, MAP3K1 L838fs PRESENT Mastopathy: PIK3CA H1047R ABSENT, MAP3K1 L838fs ABSENT	Tumor: MAP3K1 R273fs PRESENT Mastopathy: MAP3K1 R273fs ABSENT
17		Tumor: GATA3 D336fs PRESENT Mastopathy: GATA3 D336fs ABSENT

## Discussion

This is one of the first studies using a targeted breast cancer gene panel and massive parallel sequencing on fibrocystic breast tissues that were diagnosed years before the development of subsequent invasive breast cancer in order to understand the genomic landscape of these benign breast tissue changes. By investigating a unique collective of 53 breast cancer patients, of whom benign pre-cancerous tissue was available for genetic analysis, we hoped to identify early molecular alterations important for breast cancer carcinogenesis. Unfortunately, due to fixation artifacts and inferior DNA quality, we were only able to successfully sequence mastopathy tissue, tumor tissue and control tissue from a total of 17 patients, which is certainly the greatest limitation of our study.

None of these 17 patients had any significant mutations in the mastopathy tissue, challenging the notion that early genetic alterations are regularly present in benign breast tissue prior to the development of breast cancer. Whether this absence of any mutations in the mastopathy tissue is due to a lack of genetic alteration or due to sample size limitation or impaired DNA quality, we cannot say with confidence. We detected three putative mutations in the mastopathies, but these mutations were either low VAF or were possibly fixation artifacts. On the other hand, in the five cases in which hotspot *TP53* or *PIK3CA* mutations were detected in the tumor, none of them was detected in the mastopathy despite an average depth of 2516x in the samples.

*PIK3CA* and *TP53* constitute two driver genes that are recurrently altered at high frequency (up to 30%) in invasive breast cancers (21). Moreover, *PIK3CA* mutations are enriched in ER + tumors (up to 45%), which corresponds to the ER+ status of all five tumors with identified hotspot mutations in our study. The mutations identified in the tumor tissue thus fit nicely into the known mutational landscape of breast cancer.

In light of our results we have to assume that benign breast tissue with fibrocystic changes does not routinely harbor any oncogenic mutations, in contrast to premalignant lesions such as ADH, DCIS, and LCIS.

A recent study by Rohan et al. also investigated the occurrence of somatic mutations in benign breast tissue of 218 patients who later developed invasive breast cancer (22). While identifying a total of 504 somatic mutations in the benign breast tissue, they found no significant difference in the overall mutation burden when compared to patients who did not develop cancer. Importantly however, the authors included proliferative lesions with atypia (ADH, ALH) into their benign breast lesions, which could account for the high number of somatic mutations detected in their collective. ADH and ALH both have 3- to 5-fold higher risk for subsequent invasive breast cancer compared to women with non-proliferative breast lesions (23) and are known to harbor genetic alterations (24, 25). Moreover, ADH is also considered to be a direct but non-obligate precursor to carcinoma (26).

Rohan et al. also compared the benign breast biopsies with tissue samples from the subsequent ipsilateral invasive breast cancer in 7 patients, and, in accordance with the results of our study, were not able to identify any shared mutations between the benign and the cancer tissue (22). One explanation for this is that since the sequenced mastopathy tissue was removed years earlier in our patients, it did not give rise to the subsequent invasive cancer. The cancer most probably developed through precursor lesion in adjacent breast tissue, or, in case of contralateral invasive carcinomas, in the tissue of the contralateral breast, which was not initially sampled.

Taking into consideration the negative results of our study, it thus remains unclear why women with fibrocystic breast disease have a statistically significant increased risk of breast cancer. Further analyses of these fibrocystic changes, perhaps on the level of gene expression, could help to clarify the role that these benign alterations play in the development of breast cancer and help to identify women with greater risk of developing subsequent invasive cancer.

## Data Availability

The datasets generated for this study can be found in European Genome-phenome Archive (EGA), EGAS00001003563.

## Ethics Statement

This study was conducted in concordance with institutional patient safety laws and has been approved by the Ethikkommission Nordwest- und Zentralschweiz (EKNZ, proposal number 2014-397). This study was performed in accordance with the Declaration of Helsinki.

## Author Contributions

SS designed and planned the project, identified the patients, created the database, and wrote part of the manuscript. LC and VP retrieved the slides and blocks from the archive, prepared the tissue, and performed the sequencing. SP and CN designed the gene panel, supervised the sequencing, analyzed the sequencing data, and wrote part the manuscript. WW gave continuous input and critically reviewed the manuscript. SM designed, planned and supervised the project, reviewed all histological slides, identified the regions of interest for sequencing, and wrote part of the manuscript.

### Conflict of Interest Statement

The authors declare that the research was conducted in the absence of any commercial or financial relationships that could be construed as a potential conflict of interest.
